# Effect of temperature and relative humidity on the effectiveness of graphene on stored product insects

**DOI:** 10.1007/s11356-025-36899-y

**Published:** 2025-09-03

**Authors:** Evagelia Lampiri, Dusan Losic, Christos G. Athanassiou

**Affiliations:** 1 School of Agricultural Sciences, University of Thessaly, Phytokou Str Nea Ionia, Magnesia, Volos, 38446 Greece; 2 School of Chemical Engineering, The University of Adelaide, Adelaide, SA 5005 Australia

**Keywords:** Graphene, Nanopesticides, Stored product insects, Temperature, Relative humidity, Natural insecticides

## Abstract

This investigation assessed the insecticidal efficacy of two graphene formulations (Gr1 and Gr2) on wheat kernels against adults of *Sitophilus oryzae* (L.) (Coleoptera: Curculionidae) and *Oryzaephilus surinamensis* (L.) (Coleoptera: Silvanidae) in relation to temperature and relative humidity (RH) at concentrations of 500 and 1000 ppm. These bioassays were conducted in all possible combinations of three temperature levels: 20, 25, and 30 °C, as well as two relative humidity levels (55 and 75%). Progeny production was also evaluated 65 days later. The species that emerged as the most susceptible to graphene-treated wheat kernels was *O. surinamensis* compared to *S. oryzae*, regardless of temperature, RH, dose, and graphene formulation. At high RH levels, increasing temperature led to a decrease in mortality of *S. oryzae* in both Gr1 and Gr2, whereas at low RH levels, the results were dependent on the dosage of graphene formulation. At 75% RH, mortality ranged at significantly lower rates compared to their corresponding ones at 55% RH, while RH appeared to have no effect at 20 °C regardless of graphene formulation. Total inhibition of the emergence of progeny production was recorded for *O. surinamensis*, while graphene failed to suppress the progeny of *S. oryzae.* The current study’s findings offer evidence supporting the feasibility of using graphene as an alternative to chemical-based pest control approach for protecting grain stored products.

## Introduction

K.S. Novoselov and A.K. Geim of the University of Manchester in the UK discovered graphene, a two-dimensional (2D) honeycomb-like lattice of single layers of C-atoms, in 2004 (Novoselov et al. 2004). For their discovery, they were granted the 2010 Nobel Prize in Physics (Geim and Novoselov^ 2007^). Graphene has many outstanding ranges of properties including structural, chemical, mechanical, optical, thermal and electrical, properties (Rao et al.^ 2009^), which are used for the application across broad sectors and successfully translated in several hundred different products and devices.

The graphene materials were explored for a range of uses such as the field of pest management for agricultural insects (Sharma et al. 2017; Wang et al. 2019a, b; Chen et al. 2022; Li et al.^ 2022^), public health insects (Dziewięcka et al. 2016; Murugan et al. 2017) but also stored grain insects (Batool et al. 2020, (Batool, et al., 2021); Moisidis et al. 2022; Lampiri et al. 2024). In most of these applications, graphene is used in depression combined with conventional pesticides. Indicatively, Li et al. (2022) conducted laboratory bioassays with a mixture of graphene oxide with four insecticides, namely, chlorantraniliprole, beta-cypermethrin, methoxyhydrazide, and spinetoram against the fall armyworm, *Spodoptera frugiperda* J.E. Smith (Lepidoptera: Noctuidae) and highlighted that compared to insecticides alone, the efficacy of the graphene oxide (GO) mixtures increased from 1.54 to 2.53 times.

Recently, Moisidis et al. (2022) utilized graphene in the form of powders to prevent post-harvest infestations caused by stored product pests showing considerable insecticidal properties. Precisely, in the study by Moisidis et al. (2022), two graphene types were tested for their ability to cause adult mortality at the rice weevil, *Sitophilus oryzae* (L.) (Coleoptera: Curculionidae)*,* the lesser grain borer, *Rhyzopertha dominica* (F.) (Coleoptera: Bostrychidae), and the red flour beetle, *Tribolium castaneum* (Herbst) (Coleoptera: Tenebrionidae). After 21 days of exposure to wheat treated at 1000 ppm, all insect species examined showed 100% mortality. The abiotic environmental variables, which prevail in storage facilities of grains and agricultural commodities, such as temperature and relative humidity (RH), are key components for the design and implementation of an integrated management strategy of stored product insects (Snelson 1987; Hagstrum and Milliken 1988; Muir 2000; Fields and Korunic 2000). For the vast majority of insecticides used in such facilities, increasing temperature has a positive effect on insect mortality (Arthur et al. 2004; Nayak and Collins 2008). Kavallieratos et al. (2021) indicated that higher mortality rates of the yellow mealworm, *Tenebrio molitor* (L.) (Coleoptera: Tenebrionidae) were caused by temperature increases from 20 to 25 and 20 to 30 °C when exposed to barley sprayed with 5 mg/kg of the organophosphate, pirimiphos-methyl. Similarly, for spinetoram, significantly increased mortality of *S. oryzae* was recorded at increased temperatures. However, pyrethroids have been found to be ineffective at elevated temperatures (Thaung and Collins 1986; Boukouvala, et al., 2022). Particularly, the study of (Boukouvala, et al. 2022) reported that both adults and larvae of *S. oryzae* and the confused flour beetle, *Tribolium confusum* Jaquelin Du Val (Coleoptera: Tenebrionidae) were more susceptible at 20 °C than at 25 and 30 °C when they were exposed to pyrethroid, etofenprox.

Nevertheless, in the case of RH, it has been established for most of the active ingredients, that its increase reduces their effectiveness and therefore their ability to control stored product insects, due to the degradation they can cause or through reducing the insect’s contact with the toxic substance. For example, Kavallieratos et al. (2021) reported that both adults and larvae of *T. molitor* exhibited higher mortality rates at 55% RH than at 75% RH when they were exposed to barley treated with pirimiphos-methyl. Interestingly, RH in conjunction with temperature has often offered conflicting results, which are related to the active ingredient, dose, and target insect species (Fields and Korunic 2000; Fang and Subramanyam 2003). Several reports have examined the effect of combining different temperature and RH levels on the efficacy of inert materials (Aldryhim 1990; Aldryhim, 1993; Korunic 1998; Fields and Korunic 2000; Subramanyam and Roesli 2000; Arthur 2000a, b, Arthur, 2001; Vayias and Athanassiou 2004; Athanassiou et al. 2007; Baliota et al. 2022). In general, increasing temperature and decreasing RH have demonstrated a positive impact on the control of insects of stored products, with some discrepancies, which may be due to the origin and formulation of inert material, dose, exposure interval, and insect species (Fields and Korunic 2000). Still, no available research reports regarding the impact of these major abiotic elements on the effectiveness of the insecticidal properties of graphene. Furthermore, although graphene has demonstrated promising insecticidal properties against various pests (Batool et al.^ 2020^; Moisidis et al. 2022; Lampiri et al. 2024), its application in grain storage raises important questions regarding food safety. The graphene materials based on many studies are generally considered as non-hazardous materials (Kostarelos et al. 2009). The cell toxicity studies reveal that their toxicity and potential biological impact depends on factors such as purity, particle size, and concentration (Farivar et al. 2021; Dziewięcka et al. 2016). For example, Dziewięcka et al. (2016) demonstrated that the toxicity of graphene oxide to the house cricket, *Acheta domesticus* (L.) (Orthoptera: Gryllidae) varied depending on material purity and exposure duration, while Farivar et al. (2021) emphasized the importance of quality control to avoid contaminants in graphene materials intended for biological applications. Currently, there is limited information on potential risks associated with its direct use on food commodities, highlighting the need for further toxicological evaluations before large-scale adoption in stored product protection.

This study aimed to evaluate how temperature and RH affect the insecticidal potential of graphene powders for the control of major stored product insects by performing an assortment of lab bioassays with two graphene powder formulations with nanosized particles, against two insect species, *S. oryzae* and the saw-toothed grain beetle, *Oryzaephilus surinamensis* (L.) (Coleoptera: Silvanidae). To achieve this, we employed a wide variety of combinations of abiotic parameters (temperature from 20 to 30 °C and RH from 55 to 75%), which are appropriate for insect growth in bulked grain commodities, while graphene efficacy was also evaluated on the suppression of the progeny production 65 days later.

## Materials and methods

### Graphene dusts

Industry-produced graphene dusts labeled Gr1 and Gr2 with nanoscale particle size, supplied by an Australian graphene manufacturer, were used as supplied for this study without further modification. The graphene powders with measured mass to provide concentrations of 500 and 1000 ppm in grain were applied to two selected surface grain insects for the bioassay study.

### Graphene powder characterization

The basic standard techniques proposed by Lampiri et al. (2024) were followed to characterize the graphene powders used in the present work. In brief, graphene samples were analyzed via transmission and scanning electron microscopes to determine their morphology and particle size. Vibrational properties and structural defeats were identified utilizing a Raman spectrometer. GR2M dispersion was drop-cast onto a Si wafer to form the samples. To determine the chemical composition, X-ray photoelectron spectroscopy was performed. The study employed an X-ray powder diffraction survey to examine crystallinity and interlayer spacing. In addition, pressed graphene sheets were subjected to contact angle measurements to determine their interfacial properties which can be related to adhesion to insect body and grain surface.

### Tested insects and commodity

The species tested were adults of *S. oryzae* and *O. surinamensis*. At the Laboratory of Entomology and Agricultural Zoology, Department of Agriculture, Crop Production, and Rural Environment, University of Thessaly, *S. oryzae* was reared on whole soft wheat kernels, while *O. surinamensis* on oat flakes, and both of them maintained in incubators that were set at 26 °C, 65% relative humidity, and constant darkness. Untreated and uninfested soft wheat kernels served as commodities for the tests. Before the experiment, a Multitest moisture meter (GODE Co, Le Catelet, France) was used to determine the wheat’s moisture content, which was found to be 11.1%.

### Bioassays

Three temperature settings (20, 25, and 30 °C) and two relative humidity levels (55 and 75%) were used in separate bioassays. As recommended by Vayias and Athanassiou (2004), saturated salt solutions were used to attain the necessary relative humidity values, while incubator chambers were used to maintain suitable temperature levels. Glass jars with a 1-L capacity (15 cm in diameter, 35 cm in height, Bormioli Rocco, Italy) were filled with 500 g of the soft wheat for the bioassays. The jars were treated with either 500 or 1000 ppm, which corresponds to 500 and 1000 mg of graphene per kg of grain, with a different series of jars for each graphene formulation and dosage. To ensure that the graphene was evenly distributed throughout the grain mass, the jars were then carefully shaken by hand for 1 min after being securely covered with lids. As controls, a different set of jars with untreated wheat kernels were utilized. Three cylindrical plastic vials (3 cm in diameter, 8-cm height, Rotilabo Sample tins Snap on cap, Carl Roth, Germany) were then filled with three samples of 20 g of the treated grain from each jar. Each vial was then filled with 20 adults of each species, using a distinct set of vials for each insect. Each combination of insect species, graphene formulation, dosage, temperature, and relative humidity underwent the whole procedure twice (i.e., six vials per combination). To achieve the desired temperature and relative humidity, the vials were put in different chambers. Insect mortality was assessed following exposure to the treated commodity for 7, 14, and 21 days. After the exposure period ended, all adults—dead or alive—were taken out of the vials. The vials were then left under the same experimental conditions for a further 65 days, during which time they were opened once more, and the progeny production was measured.

### Statistical analysis

The JPM 7 software (SAS Institute Inc. Cary, North Carolina, USA) was used to perform a repeated measures multivariate analysis of variance (MANOVA-Fit with Wilk’s Lambda) for both mortality data and progeny production counts, using graphene formulation, dosage, temperature, and relative humidity as main factors. One-way ANOVA was performed for each graphene formulation, dosage, RH, exposure period, and insect species to identify the variations across the various temperatures. At *P* = 0.05, the Tukey–Kramer (HSD) test was used to separate the means. Additionally, using SPSS software version 17.0, the independent sample *t* test at *P* = 0.05 (Sokal and Rohlf 1995) was used to compare the means of various RH levels for each temperature, dosage, insect species, and exposure interval. Each insect species’ progeny production was also subjected to a one-way ANOVA with graphene formulation, dosage, and RH as the main effects. The Tukey–Kramer (HSD) test was used to separate the means at *P* = 0.05. Additionally, the independent sample *t* test was used at *P* = 0.05 to compare the progenies’ means across various RH levels for each temperature, dosage, and insect species (Sokal and Rohlf 1995).

## Results

### Characterization of structure and chemical composition of graphene powders

**Fig. 1:**
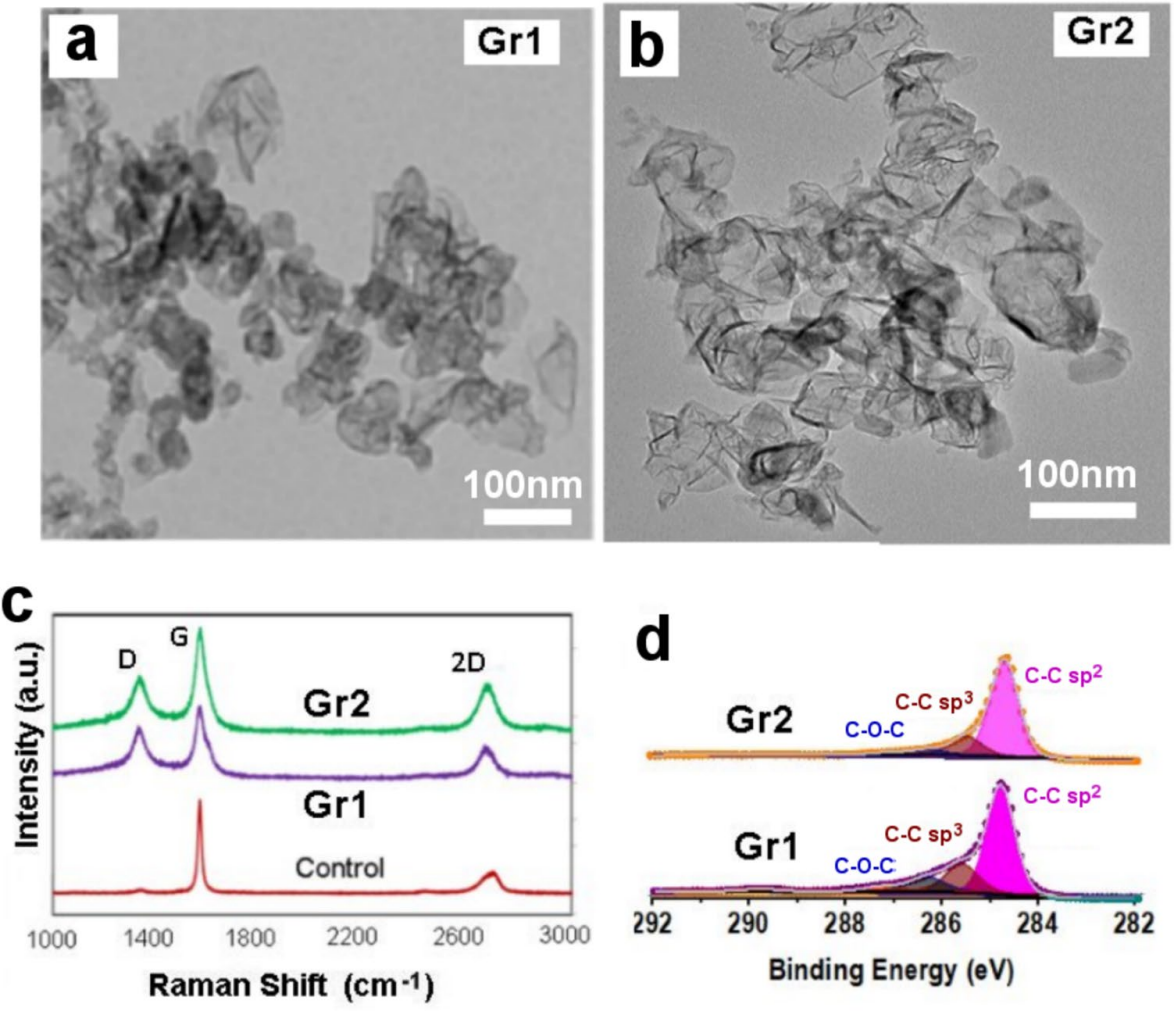
Characterization of industrially produced graphene powders showing **a**, **b** TEM image revealing graphene nanosheets morphology and structures, **c** Raman spectroscopy graphs comparing to control of few-layer graphene, and **d** XPS graph displaying the C1s carbon peak in high resolution and determining elemental chemical analysis

Their structural, interfacial, and chemical characteristics, such as lateral particle size, number of layers, degree of structural defects, crystallinity, oxygen content, surface charge, and hydrophobicity/hydrophilicity, were summarized in Fig. 1. With a bulk density of 30 mg/ml, the graphene powders appeared with a fluffy, airy, and soft feel, indicating a high surface area and lower aggregation (stacking) of graphene nanosheets. Characterization results by TEM (Fig. 1a–b) confirmed that these graphene powder materials possess a nanoflake structure, with particles that are typically 100 nm in size and a large distribution number of layers from 5 to 20. These morphological characteristics with high aspect ratio (length vs thickness) can classify this material as graphene two-dimensional (2d) nano powders. The Raman spectroscopy results presented in Fig. 1c displayed typical graphs with two distinctive bands, D and G, at around 1345 cm^−1^ and 1600 cm^−1^, respectively, and one 2D band at 2719 cm^−1^ when compared to the control (few layers graphene). Based on the peak intensity ratio of the D to G bands (ID/IG) in the range of –0.5 to 1.0, these results indicate the extent of defects, which suggests a high level of structural disorder in the carbon sp^2^ graphene structure. The XPS results (Fig. 1d) showed levels of carbon, oxygen, and Csp^2^ carbon of 95.74 C%, 4.22 O%, and 64.39 Csp^2^ for Gr 1 and 98.05 C%, 2.09 O%, and 76.50 Csp^2^ for Gr2 indicating the presence of oxygen and slight differences in chemical properties between these two graphene materials. These observed structural, compositional, and chemical characteristics are in agreement with structurally disordered graphene materials (Farivar et al. 2021). These characteristics appear to enhance insecticidal properties compared to defect-free graphene showed in the recent study (Lampiri et al. 2024), although the underlying reasons for this behavior are not yet fully understood.

### Adult mortality

While all main effects and their interactions, with the exception of dose and its interactions, affected the insect’s mortality in *O. surinamensis*, the majority of main effects and their interactions were significant for *S. oryzae* (Table 1).

**Table 1:** Repeated-measures MANOVA parameters for the mortality counts of the species tested [total degrees of freedom *(df)* = 180]

		*Sitophilus oryzae*		*Oryzaephilus surinamensis*	
	*df*	*F*	*P*	*F*	*P*
All between variables	35	56.1	< 0.01	756.7	< 0.01
Intercept	1	3532.9	< 0.01	81,667.9	< 0.01
Graphene formulation (F)	2	707.8	< 0.01	13,146.2	< 0.01
Dose (D)	1	43.0	< 0.01	0	1.00
Temperature (T)	2	26.4	< 0.01	13.1	< 0.01
Relative humidity (RH)	1	199.1	< 0.01	5.8	< 0.01
F × D	2	22.7	< 0.01	0.8	0.41
F × T	4	9.6	< 0.01	17.1	< 0.01
F × RH	2	30.6	< 0.01	5.1	< 0.01
D × T	2	0.1	0.88	0.06	0.94
D × RH	1	2.8	0.09	0.8	0.35
T × RH	2	29.0	< 0.01	10.5	< 0.01
F × D × T	4	2.3	0.05	0.6	0.60
D × T × RH	2	5.1	< 0.01	0.4	0.62
F × T × RH	4	3.4	< 0.01	12.4	< 0.01
F × D × RH	2	2.5	0.07	1.5	0.20
F × D × T × RH	4	2.4	0.04	0.7	0.57
All within interactions	70	4.9*	< 0.01	11.7*	< 0.01
Time	2	295.7*	< 0.01	231.4*	< 0.01
Time × F	4	32.5*	< 0.01	110.5*	< 0.01
Time × D	2	3.8*	0.02	2.1	0.11
Time × T	4	5.4*	< 0.01	5.9*	< 0.01
Time × RH	2	19.6*	< 0.01	17.8*	< 0.01
Time × F × D	4	4.8*	< 0.01	1.2	0.28
Time × F × T	8	2.6*	< 0.01	5.2*	< 0.01
Time × F × RH	4	4.9*	< 0.01	16.0*	< 0.01
Time × D × T	4	2.6*	0.03	0.9	0.42
Time × D × RH	2	6.1*	< 0.01	1.7	0.18
Time × T × RH	4	2.0	0.09	11.6 *	< 0.01
Time × F × D × T	8	1.8	0.06	1.4	0.16
Time × D × T × RH	4	1.1	0.31	1.8	0.11
Time × F × T × RH	8	1.9*	0.05	10.0*	< 0.01
Time × F × D × RH	4	3.3*	0.01	1.4	0.22
Time × F × D × T × RH	8	0.7	0.65	1.8	0.06

*Wilks’ Lamda approximate *F* value

At 55% RH, the mortality rates of *S. oryzae* fluctuated at high levels even from the first 7 days of exposure to Gr1, with the highest mean numbers recorded at 20 (75%) and 30 °C (77.5%) at 500 and 1000 ppm, respectively (Table 2). Among the temperatures examined, at 55% RH and 1000 ppm of Gr1, higher mortality levels were noted at the highest temperatures (25 and 30 °C), while at 500 ppm at 20 °C at least for the first two exposure intervals (7 and 14 days), while 21 days after, the mortality rates of *S. oryzae* ranged at similar levels for all three temperatures with no significant differences to be found. On the contrary, at 75% RH, higher efficacy of Gr1 against *S. oryzae* adults was found at the lowest temperature tested at both doses throughout the post-exposure period (Table 2). At 20 °C, RH did not affect the effectiveness of Gr1, while at the higher temperatures (25 and 30 °C), significantly higher mean mortality of *S. oryzae* was recorded at 55% compared to 75% RH at both doses examined. Indicatively, the mortality at 55% RH ranged at 90 and 98.3% at 1000 ppm at 25 and 30 °C, respectively, whereas 48.3 and 58.3% were the respective percentages at 75% RH (Table 2). As in the case of Gr1, Gr2 at 55% was effective in controlling *S. oryzae* adults from the initial exposure period (7 days) at both 500 and 1000 ppm, with the highest mortality rates recorded at 30 (57.5%) and 20 °C (65.8%), respectively (Table 3). Nevertheless, in contrast to Gr1, at 55% RH, temperature did not affect the effectiveness of the graphene formulation Gr2, as no noticeable variations were found in any case (dosage or exposure interval). Similarly to Gr1, at 75% RH with the exception of 1000 ppm on the final day of exposure, where mortality at 20 and 30 °C did not differ substantially, the mortality of *S. oryzae* was often significantly greater at 20 °C than at 25 and 30 °C (Table 3). Among the different RH levels, at 55% RH, as in the case of Gr1, higher mortality rates were achieved at 25 and 30 °C compared to 75% RH, while the mortality levels of *S. oryzae* were considerably greater at 55% RH (75.8%) than at 75% RH (48.3%) on day 21 of exposure at 500 ppm, suggesting that RH did not influence the efficacy of Gr2 at 20 °C (Table 3).

**Table 2:** Mean (± SE) mortality (%) of *S. oryzae* adults exposed to 500 and 1000 ppm of graphene formulations Gr1 on wheat, at combinations of three temperatures (20, 25, and 30 °C) and two relative humidity levels (55 and 75%), at three exposure intervals (7, 14, and 21 days)

		55% RH			75% RH		
Exposure time (d)	Dose (ppm)	20 °C	25 °C	30 °C	20 °C	25 °C	30 °C
7 d	0	0.0 ± 0.0b	0.0 ± 0.0b	6.6 ± 2.1a*	0.0 ± 0.0	1.6 ± 1.0	0.0 ± 0.0
	500	75.0 ± 6.0a	55.8 ± 3.2b*	55.8 ± 2.7b*	62.5 ± 11.2a	30.8 ± 5.0b	35.0 ± 3.6b
	1000	64.1 ± 5.2	67.5 ± 3.5*	77.5 ± 2.1*	68.3 ± 7.9a	32.5 ± 3.5b	42.5 ± 4.0b
14 d	0	2.5 ± 1.7b	5.8 ± 2.7b	15.8 ± 3.2a*	4.1 ± 2.0	2.5 ± 1.7	0.83 ± 0.8
	500	85.8 ± 3.0a	68.3 ± 3.0b*	77.5 ± 4.2ab*	69.1 ± 9.9a	45.8 ± 5.8ab	41.6 ± 3.0b
	1000	77.5 ± 3.5b	85.8 ± 2.7ab*	94.1 ± 2.3a*	78.3 ± 6.1a	44.1 ± 3.2b	51.6 ± 2.7b
21 d	0	6.6 ± 2.4b	8.3 ± 2.4b	25.8 ± 2.3a*	7.5 ± 2.8	3.3 ± 1.6	2.5 ± 1.7
	500	81.6 ± 3.3	75.8 ± 4.3*	87.5 ± 4.0*	71.6 ± 10.1a	45.8 ± 5.8ab	45.0 ± 3.4b
	1000	89.1 ± 2.3b	90.0 ± 2.5ab*	98.3 ± 1.0a*	80.0 ± 6.8a	48.3 ± 4.2b	58.3 ± 5.2b

Where there are no letters, no significant variations were observed; the means within each row and RH level followed by the same lowercase letter are not statistically different (*df* = 2.17, HSD test at 0.05 in all situations). Asterisks (*) indicate that the means within each temperature and dosage are significantly different; in the absence of asterisks, no significant variations were observed (*df* = 1.11,* t* test at 0.05 in all cases)

**Table 3:** Mean (± SE) mortality (%) of *S. oryzae* adults exposed to 500 and 1000 ppm of graphene formulations Gr2 on wheat, at combinations of three temperatures (20, 25, and 30 °C) and two relative humidity levels (55 and 75%), at three exposure intervals (7, 14, and 21 days)

		55%			75%		
Exposure time	Dose	20 °C	25 °C	30 °C	20 °C	25 °C	30 °C
7 d	0	0.0 ± 0.0	1.6 ± 1.0	4.1 ± 2.7	0.0 ± 0.0	1.6 ± 1.6	0.0 ± 0.0
	500	46.6 ± 8.5	41.6 ± 8.8*	57.5 ± 7.2*	36.6 ± 3.0a	8.3 ± 2.1b	10.8 ± 1.5b
	1000	65.8 ± 6.5	50.8 ± 6.3*	51.6 ± 10.2*	75.0 ± 6.9a	14.1 ± 2.3b	34.1 ± 7.2b
14 d	0	1.6 ± 1.0	5.8 ± 2.3	10.0 ± 3.6*	2.5 ± 1.7	5.8 ± 3.2	0.8 ± 0.8
	500	66.6 ± 8.3	57.5 ± 11.3*	80.8 ± 5.8*	48.3 ± 4.4a	21.6 ± 5.1b	18.3 ± 2.7b
	1000	87.5 ± 3.8	84.1 ± 3.5*	93.3 ± 5.7*	85 ± 4.6a	30 ± 4.2c	57.5 ± 4.0b
21 d	0	3.3 ± 1.6b	12.5 ± 4.9ab	16.6 ± 3.3a*	4.1 ± 1.5	5.8 ± 3.2	0.8 ± 0.8
	500	75.8 ± 8.0*	65.8 ± 10.4*	88.3 ± 4.5*	48.3 ± 4.4a	27.5 ± 8.9ab	24.1 ± 2.3b
	1000	90.8 ± 3.7	97.5 ± 1.7*	96.6 ± 3.3*	89.1 ± 3.0a	49.1 ± 6.8b	72.5 ± 4.9a

Where there are no letters, no significant variations were observed; the means within each row and RH level followed by the same lowercase letter are not statistically different (*df* = 2.17, HSD test at 0.05 in all situations). Asterisks (*) indicate that the means within each temperature and dosage are significantly different; in the absence of asterisks, no significant variations were observed (*df* = 1.11, *t* test at 0.05 in all cases)

Regarding *O. surinamensis*, mortality in both graphene formulations (Gr1 and Gr2) was particularly higher at all exposure intervals, doses, temperatures, and RH examined compared to *S. oryzae*, as complete mortality was observed in the majority of the cases (Tables 4 and 5). Indicatively, the lowest mortality rate was recorded in Gr1 at 1000 ppm at 20 °C and 55% RH on the 7th day of exposure (99.1%), while in Gr2 at 500 ppm at 30 °C and 75% RH on the 7th day of exposure (95%). Significant differences between temperatures were noted only in the case of Gr2 at 1000 ppm and 55% RH on the 7th day of exposure, where mortality at 25 °C was significantly lower (95.8%) than that at 20 and 30 °C (100%) (Table 5), while no differences were recorded between the various RH levels in any of the graphene formulations tested (Tables 4 and 5).

**Table 4:** Mean (± SE) mortality (%) of *O. surinamensis* adults exposed to 500 and 1000 ppm of graphene formulations Gr1 on wheat, at combinations of three temperatures (20, 25, and 30 °C) and two relative humidity levels (55 and 75%), at three exposure intervals (7, 14, and 21 days)

		55% RH			75% RH		
Exposure time (d)	Dose (ppm)	20 °C	25 °C	30 °C	20 °C	25 °C	30 °C
7 d	0	3.3 ± 1.6	4.1 ± 1.5	5.0 ± 1.8	0.0 ± 0.0b	5.0 ± 2.5b	13.3 ± 2.4a*
	500	100 ± 0.0	100 ± 0.0	100 ± 0.0	100 ± 0.0	100 ± 0.0	100 ± 0.0
	1000	99.1 ± 0.8	100 ± 0.0	100 ± 0.0	100 ± 0.0	100 ± 0.0	100 ± 0.0
14 d	0	10.0 ± 2.8	14.1 ± 1.5	13.3 ± 1.6	6.6 ± 1.6b	8.3 ± 3.8b	24.1 ± 2.7a*
	500	100 ± 0.0	100 ± 0.0	100 ± 0.0	100 ± 0.0	100 ± 0.0	100 ± 0.0
	1000	100 ± 0.0	100 ± 0.0	100 ± 0.0	100 ± 0.0	100 ± 0.0	100 ± 0.0
21 d	0	20.8 ± 4.1	18.3 ± 2.1	17.5 ± 1.7	12.5 ± 1.7b	35.0 ± 6.1a*	48.3 ± 2.7a*
	500	100 ± 0.0	100 ± 0.0	100 ± 0.0	100 ± 0.0	100 ± 0.0	100 ± 0.0
	1000	100 ± 0.0	100 ± 0.0	100 ± 0.0	100 ± 0.0	100 ± 0.0	100 ± 0.0

Where there are no letters, no significant variations were observed; the means within each row and RH level followed by the same lowercase letter are not statistically different (*df* = 2.17, HSD test at 0.05 in all situations). Asterisks (*) indicate that the means within each temperature and dosage are significantly different; in the absence of asterisks, no significant variations were observed (*df* = 1.11, *t* test at 0.05 in all cases)

**Table 5:** Mean (± SE) mortality (%) of *O. surinamensis* adults exposed to 500 and 1000 ppm of graphene formulations Gr2 on wheat, at combinations of three temperatures (20, 25, and 30 °C) and two relative humidity levels (55 and 75%), at three exposure intervals (7, 14, and 21 days)

		55% RH			75% RH		
Exposure time (d)	Dose (ppm)	20 °C	25 °C	30 °C	20 °C	25 °C	30 °C
7 d	0	5.0 ± 1.8*	5.8 ± 2.3	4.1 ± 1.5	0.0 ± 0.0	8.3 ± 3.3	8.3 ± 2.4
	500	99.1 ± 0.8	95.8 ± 1.5	96.6 ± 2.4	100 ± 0.0	96.6 ± 2.4	95.0 ± 1.2
	1000	100 ± 0.0a	95.8 ± 1.5b	100 ± 0.0a	100 ± 0.0	99.1 ± 0.8	100 ± 0.0
14 d	0	9.1 ± 2.3	15.0 ± 3.1	15.0 ± 3.4	6.6 ± 2.7	12.5 ± 3.5	16.6 ± 3.8
	500	100 ± 0.0	100 ± 0.0	99.1 ± 0.8	100 ± 0.0	100 ± 0.0	98.3 ± 1.0
	1000	100 ± 0.0	100 ± 0.0	100 ± 0.0	100 ± 0.0	100 ± 0.0	100 ± 0.0
21 d	0	19.1 ± 3.2	20.8 ± 2.0	20.8 ± 2.3	12.5 ± 3.3b	24.1 ± 7.0ab	41.6 ± 3.3a*
	500	100 ± 0.0	100 ± 0.0	100 ± 0.0	100 ± 0.0	100 ± 0.0	99.1 ± 0.8
	1000	100 ± 0.0	100 ± 0.0	100 ± 0.0	100 ± 0.0	100 ± 0.0	100 ± 0.0

Where there are no letters, no significant variations were observed; the means within each row and RH level followed by the same lowercase letter are not statistically different (*df* = 2.17, HSD test at 0.05 in all situations). Asterisks (*) indicate that the means within each temperature and dosage are significantly different; in the absence of asterisks, no significant variations were observed (*df* = 1.11, *t* test at 0.05 in all cases)

### Progeny production

All main effects influenced progeny production of both *S. oryzae* and *O. surinamensis* apart from dose, but not all of their interactions (Table 6). Progeny production of *S. oryzae* ranged at high levels at all doses and graphene formulations tested (Tables 7 and 8). Twenty degrees centigrade had a significantly lower number of progenies compared to 25 and 30 °C for both graphene formulations and RH levels, while among the RH levels examined, at 75% RH, a significantly higher number of progenies was recorded at all temperatures for Gr2 and for Gr1 at 25 and 30 °C. In contrast, progeny production of *O. surinamensis* was zero regardless of graphene formulation, dose, temperature, and RH tested (Tables 7 and 8).

**Table 6:** MANOVA parameters for the progeny production counts of the species tested [total degrees of freedom *(df)* = 180]

		*Sitophilus oryzae*		*Oryzaephilus surinamensis*	
	*df*	*F*	*P*	*F*	*P*
All between variables	35	36.6	< 0.01	47.9	< 0.01
Intercept	1	10,531.5	< 0.01	630.5	< 0.01
Graphene formulation (F)	2	48.7	< 0.01	630.2	< 0.01
Dose (D)	1	0.2	0.64	0.7	0.37
Temperature (T)	2	419.0	< 0.01	3.2	0.04
Relative humidity (RH)	1	244.2	< 0.01	107.0	< 0.01
F × D	2	0.6	0.54	0.7	0.46
F × T	4	6.7	< 0.01	3.2	0.01
F × RH	2	2.5	0.08	107.1	< 0.01
D × T	2	2.8	0.05	0.3	0.67
D × RH	1	0.06	0.80	3.5	0.06
T × RH	2	6.6	< 0.01	9.6	< 0.01
F × D × T	4	4.3	< 0.01	0.4	0.80
D × T × RH	2	0.6	0.53	0.8	0.44
F × T × RH	4	4.3	< 0.01	9.6	< 0.01
F × D × RH	2	2.7	0.06	3.5	0.03
F × D × T × RH	4	2.3	0.05	0.8	0.52

**Table 7:** Mean progeny production (number/vial ± SE) for *S. oryzae* and *O. surinamensis*, 65 days after the removal of the parental individuals, in graphene formulation Gr1-treated grains

		55% RH			75% RH		
Insect species	Dose (ppm)	20 °C	25 °C	30 °C	20 °C	25 °C	30 °C
*S. oryzae*	0	227.5 ± 17.0c	334.5 ± 7.8b	395.6 ± 12.3a	314.5 ± 11.6b*	483.8 ± 9.5a*	492.3 ± 16.8a*
	500	149.0 ± 27.5b	353.3 ± 10.5a	364.8 ± 21.3a	179.5 ± 7.9b	455.5 ± 19.0a*	423.0 ± 6.9a*
	1000	178.5 ± 22.6b	359.0 ± 10.1a	286.1 ± 35.4a	215.1 ± 18.8b	438.1 ± 28.2a*	465.3 ± 21.4a*
*O. surinamensis*	0	55.1 ± 9.1a	62.5 ± 5.1a	15.8 ± 3.1b	94.8 ± 9.9*	134.6 ± 22.5*	141.5 ± 10.4*
	500	0.1 ± 0.1	0.0 ± 0.0	0.0 ± 0.0	0.0 ± 0.0	0.0 ± 0.0	0.0 ± 0.0
	1000	0.0 ± 0.0	0.0 ± 0.0	0.0 ± 0.0	0.0 ± 0.0	0.0 ± 0.0	0.0 ± 0.0

Where there are no letters, no significant changes were observed; the means within each row and RH level followed by the same lowercase letter are not statistically different (*df* = 2.17, HSD test at 0.05 in all situations). Asterisks (*) indicate significant differences in means within each insect species, temperature, and dosage; in the absence of asterisks, no significant differences were observed (*df* = 1.11,* t* test at 0.05 in all cases)

**Table 8:** Mean progeny production (number/vial ± SE) for *S. oryzae* and *O. surinamensis*, 65 days after the removal of the parental individuals, in graphene formulation Gr2-treated grains

		55% RH			75% RH		
Insect species	Dose (ppm)	20 °C	25 °C	30 °C	20 °C	25 °C	30 °C
*S. oryzae*	0	218.5 ± 11.0b	359.5 ± 8.5a	391.0 ± 7.9a	320.1 ± 9.2b*	483.0 ± 10.8a*	483.6 ± 11.5a*
	500	134.3 ± 28.4b	323.0 ± 6.0a	258.3 ± 46.9a	230.1 ± 8.7c*	404.1 ± 13.1b*	482.5 ± 11.2a*
	1000	70.6 ± 17.9c	384.0 ± 14.8a	280.8 ± 34.9b	158.6 ± 24.4b*	443.8 ± 19.1a*	416.5 ± 22.8a*
*O. surinamensis*	0	58.1 ± 5.4ab	67.1 ± 11.1a	27.6 ± 12.0b	96.3 ± 11.4*	107.1 ± 12.9*	113.8 ± 8.5*
	500	0.0 ± 0.0	0.0 ± 0.0	0.0 ± 0.0	0.0 ± 0.0	0.0 ± 0.0	0.0 ± 0.0
	1000	0.0 ± 0.0	0.0 ± 0.0	0.0 ± 0.0	0.0 ± 0.0	0.0 ± 0.0	0.0 ± 0.0

Where there are no letters, no significant changes were observed; the means within each row and RH level followed by the same lowercase letter are not statistically different (*df* = 2.17, HSD test at 0.05 in all situations). Asterisks (*) indicate significant differences in means within each insect species, temperature, and dosage; in the absence of asterisks, no significant differences were observed (*df* = 1.11, *t* test at 0.05 in all cases)

## Discussion

The current study’s findings showed that in comparison to adults of *S. oryzae*, *O. surinamensis* was extremely sensitive to both graphene formulations, as complete mortality was observed already from the first observation in all combinations of temperature, RH, dose, and graphene formulation. Evidence that *O. surinamensis* is among the insect species most sensitive to inert substances has been reported (Fields and Korunic 2000). For instance, Mohitazar et al. (2009) found greater sensitivity of this species compared to *T. castaneum* when exposed to the diatomaceous earth (DE) formulation SilicoSec. Soft-bodied species such as *O. surinamensis* are generally more vulnerable to inert materials than harder-bodied species like *Tribolium* spp. (Arthur 2000a, b; Vayias and Athanassiou 2004), likely due to easier cuticle penetration and subsequent desiccation (Fields and Korunic 2000).

The effectiveness of inert materials is often enhanced at higher temperatures due to increased water loss and desiccation stress (Fields and Korunic 2000; Subramanyam and Roesli 2000; Athanassiou et al. 2005; Losic and Korunic 2018). Higher temperatures may also promote insect movement, potentially increasing contact with dust particles (Fields and Korunic 2000). Our results align partially with this, as *S. oryzae* mortality at 1000 ppm of Gr1 and 55% RH was higher at 25 and 30 °C than at 20 °C. However, at 75% RH, mortality was instead higher at 20 °C. Similar observations have been reported. Indicatively, Fields and Korunic (2000) noted higher *S. oryzae* mortality at 20 °C with certain DEs. Likewise, Aldryhim (1990) found reduced efficacy of Dryacide at elevated temperatures against *T. confusum*. In our study, temperature had only a minor effect on *S. oryzae* mortality with Gr2 at 55% RH, which is similar to the findings of Athanassiou et al. (2007), who reported consistent mortality across temperatures at 55% RH with DEA-P—a formulation consisting of diatomaceous earth containing 0.3% crystalline silica and abamectin (0.25% active ingredient)—against the larger grain borer, *Prostephanus truncatus* (Coleoptera: Bostrychidae). These patterns suggest that temperature effects on graphene efficacy may be influenced by other variables such as RH and formulation characteristics (Fields and Korunic^ 2000^; Vayias and Athanassiou 2004).

Concerning RH, increasing it from 55 to 75% generally reduced graphene efficacy against *S. oryzae*, especially at 25 and 30 °C. This observation is consistent with numerous studies on inert dusts (Aldryhim 1993; Korunic 1998; Arthur 2000a,b, 2001; Fields and Korunic 2000; Vayias and Athanassiou 2004; Baliota et al. 2022). Vayias and Athanassiou (2004) found *T. confusum* to be more susceptible to DE at 55% compared to 65% RH. At higher RH, insects retain moisture more effectively and reduce cuticular desiccation (LePatourel 1986; Mewis and Ulrichs 2001; Subramanyam and Roesli 2000). Additionally, under humid conditions, inert materials may absorb atmospheric moisture, leading to particle saturation and reduced efficacy (Arthur 2001; Stathers et al. 2004). In our case, RH had minimal impact at 20 °C, consistent with Baliota et al. (2022), who also reported comparable mortality at 15 and 20 °C regardless of RH. These findings suggest that under cooler conditions, temperature stress may outweigh RH effects.

Both graphene formulations were effective in controlling adults of both species. Our earlier work (Moisidis et al. 2022; Lampiri et al. 2024) reported similar outcomes, with 100% mortality of *S. oryzae* and *R. dominica* at 500 and 1000 ppm within 7 days of exposure. Lampiri et al. (2024) also demonstrated that efficacy varied depending on graphene particle size, insect species, and dose. In the present study, differences in temperature effects were observed between Gr1 and Gr2 at 55% RH for *S. oryzae*, suggesting that formulation-specific properties influence graphene performance, consistent with variability seen in other inert materials (Fields and Korunic 2000). Progeny production was entirely suppressed for *O. surinamensis* across all treatments, while *S. oryzae* continued to produce progenies despite high adult mortality. This is consistent with Lampiri et al. (2024), who observed high progeny numbers even when parental mortality was elevated. Moisidis et al. (2022) reported lower progeny numbers with other types of graphene, implying that progeny suppression may be influenced by the graphene type used.

In conclusion, the present findings highlight graphene’s potential as an insecticidal material for stored product protection. Temperature and RH significantly influenced its efficacy, with low RH and, in some cases, higher temperatures enhancing effectiveness. Yet, graphene remained effective under some cooler and high-RH scenarios, especially for highly susceptible species. Progeny suppression varied by species, underscoring the complexity of insect response. Overall, graphene represents a promising alternative to chemical insecticides, though further work is required to optimize its use under diverse storage conditions.

## Data Availability

Data will be provided after request.
