# Correction to: Effect of temperature and relative humidity on the effectiveness of graphene on stored product insects

**DOI:** 10.1007/s11356-025-37215-4

**Published:** 2025-11-19

**Authors:** Evagelia Lampiri, Dusan Losic, Christos G. Athanassiou

**Affiliations:** 1 School of Agricultural Sciences, University of Thessaly, Phytokou Str Nea Ionia, 38446 Volos, Magnesia Greece; 2 School of Chemical Engineering, The University of Adelaide, Adelaide, SA 5005 Australia

**Correction to: Environmental Science and Pollution Research (2025) 32:21243–21252**

10.1007/s11356-025-36899-y

The article Effect of temperature and relative humidity on the effectiveness of graphene on stored product insects written by Evagelia Lampiri, Dusan Losic and Christos G. Athanassiou was originally published under exclusive license to ©The Author(s), under exclusive licence to Springer-Verlag GmbH Germany, part of Springer Nature. As a result of the subsequent decision to publish the article under the open access model, the article’s copyright notice was changed on 5 November 2025 to ©The Author(s) 2025 and the article is now distributed under a Creative Commons Attribution 4.0 International License, which permits use, sharing, adaptation, distribution and reproduction in any medium or format, as long as you give appropriate credit to the original author(s) and the source, provide a link to the Creative Commons licence, and indicate if changes were made. The images or other third party material in this article are included in the article's Creative Commons licence, unless indicated otherwise in a credit line to the material. If material is not included in the article's Creative Commons licence and your intended use is not permitted by statutory regulation or exceeds the permitted use, you will need to obtain permission directly from the copyright holder. To view a copy of this licence, visit http://creativecommons.org/licenses/by/4.0/.

The original article has been corrected.

